# Hurricane María and Public Health in Puerto Rico: Lessons Learned to Increase Resiliency and Prepare for Future Disasters

**DOI:** 10.5334/aogh.3869

**Published:** 2022-09-23

**Authors:** Gabriela R. Guerra Velázquez

**Affiliations:** 1 Boston College, MD Candidate Class of 2025, Ponce Health Sciences University, PR

**Keywords:** Hurricane Maria, Public Health, Infrastructure, Climate, Future

## Abstract

**Background:**

On September 20, 2017, Hurricane Maria, a devastating Category 5 storm struck the Caribbean Island of Puerto Rico and officially took the lives of 2 975 people although the Harvard University survey in 2018 placed that number much higher at 4 645 [1,2]. The island’s infrastructure was devastated. Eight months later in May 2020, many vital services including telecommunications, utilities, and health care systems had not yet been repaired.

**Objectives:**

To (1) review the immediate public health problems and the longer-term repercussions of Hurricane Maria; (2) identify pre-existing infrastructural deficiencies, health disparities, and problems in governance that may have increased vulnerability and delayed recovery; and (3) offer proposals for preventive measures to increase resiliency and adequately prepare Puerto Rico for future disasters.

**Methods:**

Data from the CDC and the Puerto Rico’s Health Department were collected and analyzed. Government publications, news articles, scholarly journal entries and previous research were examined. Interviews were conducted with local citizens and public health professionals. The author’s personal experience is referenced.

**Findings:**

The Puerto Rican Electric Power Authority (PREPA) and the Puerto Rico Aqueduct and Sewer Authority (PRASA) both had severely weakened infrastructures before the hurricane as a result of a massive financial crisis that had begun in 2006. These pre-existing weaknesses increased vulnerability and made reconstruction more challenging. Approximately 95% of the cell towers in Puerto Rico sustained significant damage during the hurricane and resulted in almost total loss of cell phone communication [3]. Subpar management of relief efforts by both federal agencies and the local government further hindered recovery, resulting in mass emigration of Puerto Ricans. The public health problems of Hurricane Maria continue to plague Puerto Rico’s citizens and will have long-term consequences.

**Conclusion:**

Lack of resilience in Puerto Rico’s infrastructure and government agencies rendered the island highly vulnerable to the detrimental effects of Hurricane María. Improvements to infrastructures and a transition towards a more sustainable way of life could improve Puerto Rico’s preparation and response to future disasters – natural and human-made.

## Introduction

Puerto Rico is an unincorporated territory of the United States of America. This island of about 9 000 *km*^2^ with a population of around 3 million people is situated in the northeast Caribbean Sea. Puerto Rico experiences the Atlantic hurricane season each year from June through November. As the world continues to experience global warming, Puerto Rico, with 66.7% of their population residing in coastal areas, will likely face more intense and frequent hurricanes [4].

In September 2017, Puerto Rico was struck by two major hurricanes in the span of two weeks. On September 6th, Hurricane Irma passed near Puerto Rico causing $1 billion in damage and three deaths. Soon after, on September 20th, Hurricane Maria struck the weakened island directly. With winds of up to 175 mph, it was the largest hurricane to have hit Puerto Rico since 1928 and the costliest in Puerto Rican history, causing estimated damages of $100 billion [5]. Not only did it have devastating economic costs, thousands of people died as a result of Hurricane Maria. The death toll was originally reported as 64 and has been revised by Governor Ricardo Rosello to 2 975 but the 2018 Harvard survey study estimated that perhaps as many as 4 645 fatalities had occurred [2].

This report will examine the immediate and delayed public health consequences of Hurricane Maria in Puerto Rico. It will explore the factors underlying the reported discrepancies in mortality. It will analyze how an infrastructure that had been damaged as the result of a previous financial crisis and by problems of governance, increased the island’s vulnerability and slowed recovery.

## Methods

### Data Collection

News articles from *The New York Times, The Washington Post, The Guardian, El Vocero* and other reliable news sources were gathered and read in detail. Government publications such as the 2017 Hurricane Season FEMA After-Action Report; the U.S. Energy Information Administration and the U.S. Census Bureau reports were thoroughly examined to obtain information and data about the infrastructure of Puerto Rico. Scholarly journal articles and research from the Milken Institute School of Public Health in collaboration with the University of Puerto Rico Graduate School of Public Health and the Yale School of Medicine were studied to understand the effects of the hurricane and the official death toll. CDC Morbidity and Mortality reports as well as CDC blog recommendations for enhanced communication were collected and analyzed. In all, about 60 of these government publications, news articles, scholarly journal entries and previous research were examined in depth to effectively understand the infrastructure of Puerto Rico before the hurricane, and to study its aftermath.

### Interviews

Leading public health professionals such as Dr. José Cordero, former Dean of the School of Public Health at the University of Puerto Rico, and Co-Director of the Puerto Rico Test site for Exploring Contamination Threats (PROTECT) and Center for Research on Childhood Exposure and Development Projects (CRECE), Dr. Gredia Huerta-Montañez, a pediatrician and researcher for PROTECT and CRECE were interviewed regarding the health preparedness of Puerto Rico and the status of public health after the hurricane [6,7]. The Director of FEMA Interpreters, Anna Canaparo, was interviewed to learn more about the relief efforts conducted by FEMA after Hurricane María [8]. Twenty Puerto Rican residents who had experienced the hurricane first-hand in different parts of the island were interviewed to gather information about what it was like to live through the natural disaster. Ten interviews of Puerto Ricans who were living outside the island were conducted. These, as well as the insights of the author’s personal experience provided the perspective of Puerto Ricans who were living on the U.S. mainland during this crisis.

## Findings

### Pre-existing infrastructural deficiencies

A significant precursor event that occurred roughly a decade before the hurricane and paved the way for the disaster was a major restructuring of Puerto Rico’s economy. The island’s economy had been based on manufacturing because a section of the Internal Revenue Code had allowed special advantages to corporations operating on the island. In 2006 this tax exemption was abolished, resulting in the collapse of the manufacturing sector, which in turn caused the island to go into a deep recession. In 2016, a federal law, the Puerto Rico Oversight, Management, and Economic Stability Act (PROMESA) was passed with the intent of alleviating the government-debt crisis and restructuring its massive debt. However, the debt restructuring plan was poorly designed and caused cuts in services and infrastructure. Consequently, the government continued to borrow more money until the debt burden became unsustainable.

A component of the island’s infrastructure that was severely affected by the economic downturn was the Puerto Rican Electric Power Authority (PREPA). The president of Advantage Business Consulting, Vicente Feliciano, explained that PREPA had limited options to remain afloat. Among those options were to increase the electricity rates, which were already high; stop paying bondholders, or abandon the maintenance of its plants. PREPA management chose the last option leading to the shutdown of several of its electricity generating plants. The system now had limited surge capacity and less ability to restore the power after the hurricane. The U.S. Army Corps of Engineers’ head of power grid restoration observed that there were elements that had not been replaced in years leaving Puerto Rico lacking both power plants, and reconstruction of the grid itself.

In addition to the electric grid, multiple other components of Puerto Rico’s infrastructure were deteriorating because of the lack of monetary resources. Before the hurricane, Puerto Rico had closed about 184 public schools because of the economic crisis. The Puerto Rico Highways and Transportation Authority (PRHTA) was starved for investments, leaving roads and bridges without the appropriate maintenance. People’s homes were not as safe and structurally stable as expected, because “before the storm, the island could afford only five building code inspectors, for a population of 3.5 million people [9].” All these factors contributed to the devastation that followed the hurricane and magnified its repercussions.

Another deteriorating component of the island’s infrastructure was its water system. In Dorado, a municipality in the northern coast of the island, the problems with water began in the 1980’s. During that time, water testing had identified industrial solvents and cleaning compounds in local wells. In the 30 years since the problems were first recognized, both Puerto Rico’s water authority and health department continued to test the water and continue finding contaminants. It was not until 2016 that the Environmental Protection Agency (EPA) declared Dorado’s water system a Superfund site. In 2017, the Natural Resources Defense Council (NRDC) reported that Puerto Rico’s systems of pipes had registered more drinking water violations than any other state or territory in the United States. About 70% of the island’s population obtains its water supply from sources that are in violation of federal health standards for drinking water [10].

The same utility that provides water also manages the sewage system; this has likely resulted in cross-contamination. In 2015, both the US Justice Department and the Environmental Protection Agency sued the PRASA because they had been dumping 6 million gallons of untreated sewage into waterways daily [11]. In multiple other municipalities, such as Salinas, on the south coast of the island, which has always been a dry area, drinking water quantity is at risk resulting in water rationing. Nearby Guayama had a coal-fired power plant that required 225 000 gallons of water per minute to circulate which also contributed to a water shortage. This plant also generated millions of tons of coal ash, which contains dangerous heavy metals including arsenic, cadmium, and mercury, contaminating the aquifers. The Agency of Toxic Substances and Disease Registry reported that some of the chemicals found in coal ash could cause cancer after continued long-term ingestion and inhalation [12].

The lack of access to clean water and the widespread use of contaminated water has many health repercussions in the Puerto Rican community. These include high rates of cancer and a high number of leptospirosis cases. A study by the Yale School of Public Health found that this potentially deadly disease was far more prevalent on the island than previously thought. The researchers, in conjunction with the CDC and PR Department of Health, had conducted serology tests on residents before the hurricane in the San Juan environs and discovered that 27% tested positive for exposure to the pathogen. These findings demonstrated that leptospirosis is a major public health problem in Puerto Rico.

The health status of the citizens of Puerto Rico reflected sharp disparities with the mainland before the hurricane, and this magnified the impact that the hurricane had on the health of the people. Even before the natural disaster, the health care system was facing challenges. About 34% of Puerto Ricans reported having fair or poor health and 15.4% of people live with disabilities, compared to the percentages of the United States being 18% and 8.6% respectively. In Puerto Rico the prevalence of diabetes is 17.2%; it is 10.5% on the mainland. The asthma rate is 12.2%; the mainland’s is 9.4%. High blood pressure occurs in 44.7% here compared to 32.3% on the mainland. The Centers for Disease Control and Prevention found that the island has one of the highest high blood pressure-related death rates among adults over 35 years of age. Overall, Puerto Ricans have a higher risk of cancer, diabetes, alcohol consumption, asthma, and infant mortality rates than other US citizens [13]. Forty-nine percent of Puerto Ricans rely on Medicaid and only 35% have employer-sponsored health insurance [14]. Access to quality health care has been hindered because of the mass migration of doctors and specialists to the U.S mainland. The American Heart Association reported that between 2014 and 2016, more than 1 400 doctors canceled their medical licenses issued in Puerto Rico. All of these are concerning public health issues by themselves, but with the addition of lack of access to insulin, the prolonged consumption of processed foods, the drinking of tainted water, and the lack of electricity that followed Hurricane Maria, it is not surprising that these pre-existing conditions increased the hurricane’s adverse effects and hindered recovery.

A major unanswered question is how to develop preventive measures that will protect public health against future disasters. Given the recent acceleration of global climate change, it is very likely that hurricanes of such magnitude will continue to occur in the coming years. If the climate continues to warm, the possibility that hurricanes will become even stronger and more destructive will increase. A 2013 study concluded that since 1975 there has been a substantial regional and global increase in the proportion of category 4 and category 5 hurricanes of 25–30% per degree Celsius of anthropogenic global warming [15]. Climate change will have a significant global impact with aggravated erosion, landslides, flooding, increased temperatures, wildfires, and many other natural disasters. The potential damage that could occur because of the combination of stronger storms, rise in sea-level and infrastructure that has continuously been exposed to these disasters is alarming.

### Acute and Delayed Aftermath

The magnitude of Hurricane María combined with the previously deteriorating infrastructure resulted in catastrophic damage to Puerto Rico. Its vast destruction devastated the power grid and left the entire island without electricity, some even 328 days, the longest blackout in United States history. The island’s communications infrastructure was also severely damaged with 85% of the 1 600 cell towers not functioning.

The collapse of the power system resulted in several immediate problems for hospitals and other health facilities. According to the US Department of Defense, “The health infrastructure was already in crisis before Hurricane María – now a majority of the island’s 69 hospitals are without electricity or fuel for generators [16].” The use of emergency generators, while helpful, was a limited substitute for electrical current because of the need for redirection of the power generated and the frequent need for maintenance and refueling. The largest private children’s medical center in the Caribbean, San Jorge Children’s Hospital, was without electricity and dependent on its generators. The generators would run out of diesel fuel multiple times during a week and the diesel fuel was not easily accessible. These generators were insufficient to power all of the essentials in a hospital such as lighting, refrigeration, emergency and operating rooms, and central air-conditioning and air flow systems; the latter is a critical need in sealed buildings with tropical climates.

Hospitals could not communicate with ambulance services or other clinics for people who needed urgent care because the power outage had disabled most cable service and telephone lines. Patients who received dialysis had not been treated in days, nebulizer treatments for asthmatics were not working because patients lacked electric services, and diabetics did not have working refrigerators to properly store their insulin. Doctor’s offices and walk-in clinics were not open initially because they had no electricity and, in some cases, no accessible path to get there. A month after the hurricane only about a dozen hospitals were operating and they were mostly located in San Juan and other major cities.

The lack of electricity and the constant difficulty of finding generators or diesel fuel also resulted in increased stress which deteriorated the mental health of the citizens. People stood in long lines outside overnight in hopes of receiving some fuel for either their car or generators. Those that could afford generators and the high costs of refueling had the luxury of having power yet those who did not have the access to generators or the money to obtain them spent months without electricity. By December 2017, close to half the population of Puerto Rico was still without power, most of these in rural areas where the reconstruction was further hindered by inaccessible roads and lack of personnel.

Another negative result of the destruction of the power grid was the lack of access to clean water, as electricity is needed to power water treatment plants. Lacking clean water, people drank water from rivers or other contaminated sources. The risk of using these waters was proved by EPA’s findings of enterococcus, the bacteria responsible for diverticulitis, and the bacteria responsible for UTIs and meningitis in the freshwater of Puerto Rico [17]. Leptospirosis, a public health problem that was already present on the island worsened after the hurricane. By the end of October there were 121 cases and 4 confirmed deaths from leptospirosis since the hurricane, about twice the average cases yearly.

The intense rain resulted in major flooding throughout Puerto Rico which caused many drownings. Flooding also caused many to lose their homes. Houses were built in flood zones and the government’s decisions of removing natural water systems, such as wetlands, increase flood damage. San Isidro, a neighborhood in Canóvanas, Puerto Rico, is in a flood zone in the middle of the Atlantic hurricane belt, making it one of the most vulnerable communities in the world due to the intensifying climate crisis. Many houses have not been repaired because FEMA often conditions recovery resources on the purchase of flood insurance, which is not affordable for the citizens of this impoverished community. Many of these people find themselves with no other options but to move from their homes.

Hurricane María seemed to have a longer lasting impact on certain vulnerable groups of the population including children. A study conducted by researchers from Puerto Rico’s government agencies and universities surveyed nearly 100 000 Puerto Rican students between the 1st of February to the 29th of June of 2018, in the island’s seven education districts. This study is one of the largest attempts in the history of the United States to survey young people after a major natural disaster [18]. The researchers found that about 47.5% of children’s family’s homes were damaged and 83.9% of children saw damaged homes. Likewise, 32% of the youth experienced shortages of food and water and 16.7% of youth did not have electricity 5 to 9 months after Hurricane María. Additionally, 7.2% of the 100 000 students reported clinically significant symptoms of post-traumatic stress disorder. The children who experienced this natural disaster were left to handle the repercussions in isolation since schools were closed for months. The economic crisis which had resulted in the closure of hundreds of schools led to thousands of children suddenly leave their stable learning community for a different school. Once they adapted to being in a new school, the schools were again closed because the hurricane destroyed them or because their school lacked electricity.

The death toll caused by Hurricane María resulted in very controversial discussions between citizens, government agencies and research institutions. The most recent tally of 2 975 deaths due to the hurricane is very different from the initial count of 64 fatalities, as reported by the government of Puerto Rico. One of the factors that made this recording so difficult was the lack of awareness of appropriate death certification practices after a natural disaster. Following the hurricane, the Department of Health instituted requirements that all licensed physicians in Puerto Rico must undergo training on how to properly fill out death certificates where a hurricane was a factor in the person’s death. The reliance on paper documents instead of electronic submissions of the patient’s files led to difficulties accurately accessing all death reports and information whether related to the hurricanes or not. The number of fatalities caused by the hurricane is not limited to the immediate deaths or physical injuries caused by the hurricane such as drowning and blunt-force trauma from collapsed buildings. These also include casualties caused by the lack of treatment, medications or surgical procedures that would have been available if it were not for the lack of resources caused by the hurricane. Although the latest investigations contain different figures, they all fall within a shared range that provides greater and more reliable numbers than the initial government report.

## Discussion

Puerto Rico experienced moments of great challenge causing pain and anguish following the hurricanes. Persons residing in Puerto Rico had no other option but to be resilient to overcome and survive these difficulties. In recent years there have been hurricanes, earthquakes, floods, and more recently the COVID-19 pandemic. The impacts of all these disasters have been intensified by the island’s long economic depression and social crisis. The physical destruction caused by Hurricane María has yet to be fully restored. Other consequences such as the tragic passing of thousands of lives, the trauma of the survivors and the long-term health effects, continue to cause negative ripple effects. Puerto Rico lost 4% of its population, about 130 000 residents, to migration to the mainland reflecting the largest loss of population in the history of the island [19].

Studies on climate change point to elevated levels of greenhouse gasses that cause temperatures and sea levels to rise, which in turn results in an increase in the intensity of atmospheric phenomena. Given these findings, it is crucial to propose solutions to better prepare the island for future natural disasters that are predicted. Several of the problems involved with climate change are a result of negligent governments that do not use their power to act with policies to improve these conditions. Governments do not prioritize the scientific knowledge that is provided to them and continue to participate in activities and decisions that adversely affect the climate. Oftentimes this behavior is justified by stating that these decisions are crucial to the economic well-being of the country. However, the consequences of this short-sighted behavior highlights how poor communities continue to be marginalized and are disproportionately affected by the repercussions of climate change.

A potential solution to tackling the climate change crisis from a governmental perspective is through public policy. Specifically, policy that focuses on climate change and places emphasis on the solutions that favor both the environment and those that are more vulnerable due to social inequalities. Policy can work towards creating laws that commit to renewable energy without compromising natural systems that are essential for the harmonious functioning of our planet.

An infrastructure that played a determining role on both the preparedness and the aftermath organization was the government officials of Puerto Rico and the United States of America. Hurricanes, unlike earthquakes, volcanic eruptions, or other natural disasters, allow enough time for preparation before the phenomenon occurs. In the case of Hurricane María, there had already been preparations because Hurricane Irma had occurred 2 weeks prior. However, Irma might have made the citizens not take the proper precautions because they felt they had escaped significant damage and expected Hurricane María would have a similar impact as Irma. The government had sent much of their resources to help nearby islands that had been impacted far worse. “When María hit, there was not a single cot or tarp in FEMA warehouses. They had all been sent to the Virgin Islands in response to Hurricane Irma [20].” In the first 3 months after the storm around 70 000 people requested tarps to be installed by the Army Corps of Engineers. An investigation conducted by CNN revealed that by December 20th two-thirds of those requests were unfulfilled, not because of the lack of tarps, but because of the improper management and administration of those resources. Potential solutions to improve the preparation and management by the government for future disasters would be to hold the politicians accountable for the promises they make. Requesting transparency in their preparation process as well as that of the companies and organizations that they work with must be a minimal requirement.

Another potential solution to tackle the problems of flooding would be to prohibit the destruction of coastal wetlands and ban construction projects in estuarine systems that protect the land from floods and erosion. Using Puerto Rico’s own natural systems to improve the broken electrical infrastructure is another potential solution. Although, “solar power has been Puerto Rico’s fastest growing source of renewable generation, increasing from 0.3% of total generation in fiscal year 2015 to 1.4% in fiscal year 2020,” [21] it must be further developed at a more consistent and rapid pace in its preparedness for a future natural disaster.

Although having power during natural disasters could prove beneficial in the quality and quantity of foods that could be stored by the people, it is important to improve access to healthier foods if the disaster results in the destruction of crops and other food sources. Federal agencies and nonprofit organizations should provide communities with foods that contain adequate nutritional values. In times of survival, the quality of the food given is often overlooked, yet this could be severely detrimental particularly to vulnerable populations. Educating the public on the necessary foods they should buy when preparing for such events is crucial. Although canned foods can be helpful in situations like these, people should be encouraged to also purchase fruits and other healthier alternatives that might not require spoilage preventing storage. Within vulnerable populations, such as expectant mothers, the need for an adequate diet is crucial. There are other strategies additional to food supply preparation and accessibility that could improve preparedness for future natural disasters.

A population that should be prioritized when thinking of how to improve the preparation of the island for future disasters is children. Studies continue to show that even if a child does not suffer from extreme post-traumatic stress disorder, there is a middle type of post-traumatic stress that can have a significant role on the learning, development, and overall well-being of the child. The analysis, “Trajectories of Posttraumatic Stress in Youths After Natural Disasters” by Betty Lai and colleagues discusses the importance of early assessment and triaging of children who have lived through weather-related disasters [22]. Part of the preparation for a natural disaster should involve professionals of pediatric mental health and for them to organize how they will provide the help needed as soon after the disaster as possible. Parents and guardians could be educated on how they could serve as a stable support system for their children after the crisis.

Regarding the lack of communication, there are temporary solutions that could be very effective for allocating aid resources while providing adequate information and education. Satellite phones, which depend on satellites in orbit rather than cell towers and electricity, can be purchased with a small solar portable panel, eliminating failure in emergencies. Prior to the disaster, these phones could be allocated to a leader within each municipality so that they could always communicate between each other, emergency-aid agencies, and the central government.

A supplement to the satellite phones could be the amateur radios, also known as ham radios. Amateur radios depend on short waves and can be powered by bicycles, metal, solar chargers, or batteries. The different options available to activate the use of these signals to communicate demonstrate how effective their usage can be during emergencies when other forms of communication are ineffective. These were used during Hurricane María, but as a last resort, and lacked organization and preparation. Allocating ham radios and teaching people how to use them would serve as an essential tool for communication before, during, and after the disaster.

A third potential solution to prevent the massive breakdown of communication that occurred on the island could be the use of huge helium balloons with a hanging antenna. The United States Marine Corps uses this novel technology, which could be adapted for civilian use and could provide the people with internet and enough signal to communicate. The balloons would be stored before the natural disaster and then once the event comes to an end the balloon is sent up “using helium or hydrogen to inflate the balloon and an internal ballast system (about five pounds of sand).” [23] Captain Michael Ginn of the Marine Corps states that the balloons’ system has a battery life of about 9 hours, and it can be launched in winds up to 45 knots. Depending on wind speed, the system can easily cover hundreds of miles before it dies. These methods could prove useful to citizens as they would not have to rely on generators or wait until the electrical towers are fixed to communicate.

Puerto Rico had infrastructure weaknesses which worsened the effects of Hurricane Maria and made recovery even more challenging. Given the current climate change there is a higher risk of hurricane damage in the future and therefore the preventative measures discussed along with other tactics should be considered to improve future preparedness.
